# Patterns and Clinicopathological Features of Histologically Proven Metastases in Hepatocellular Carcinoma

**DOI:** 10.7759/cureus.69385

**Published:** 2024-09-14

**Authors:** Muhammad Zamil, Hina Maqbool, Sajid Mushtaq, Usman Hassan, Maryam Hameed, Umer Sheikh

**Affiliations:** 1 Pathology, Shaukat Khanum Memorial Cancer Hospital and Research Centre, Lahore, PAK

**Keywords:** clinicopathological features of hcc, hcc, hepato cellular carcinoma, histology of hcc, histology of metastatic hcc, metastasis, patterns of hcc

## Abstract

Hepatocellular carcinoma (HCC) is the most common primary liver malignant neoplasm. Multiple risk factors have been identified for several decades for this overly aggressive tumor. HCC is an overly aggressive malignancy with frequent intrahepatic and extrahepatic metastasis. In our practice, we have observed that HCC has the propensity to metastasize to very unusual sites and can sometimes show variable patterns leading to diagnostic difficulty. In this study of 257 patients, we aim to discuss the unusual sites of HCC metastasis, the various patterns of metastasis, clinicopathological features, and the most common cause of HCC in our population. In the course of our research study, we systematically extracted a comprehensive dataset comprising 257 instances of metastatic HCCs from the hospital database spanning the period from 2016 to February 2022. The assessment of metastatic sites uncovered a wide range of locations, reflecting significant diversity. The most common location was bone, with 135 cases (52.5%). The vertebral column was the most common location among bony metastasis, with 63 cases (24.7%). Morphologically, the most common histological pattern observed was pure trabecular in 192 patients (74.7%). All cases were diagnosed with the help of immunohistochemical stains. Out of 257 cases, 29.18% were diagnosed using glypican-3 and HepPar1, while 26.1% relied solely on HepPar1 positivity. HepPar1 was performed in a total of 240 cases, and positivity was seen in 205 cases (85.5%). In summary, our study represents the most comprehensive investigation of clinicopathological characteristics in metastatic HCC conducted within the past 20 years. It helps understand the histological and immunohistochemical features useful for diagnosis at metastatic sites for tumors with an unknown primary.

## Introduction

Hepatocellular carcinoma (HCC) is the fourth most common neoplasm leading to cancer-related deaths worldwide. Over several decades, multiple risk factors have been identified for developing this overly aggressive tumor, including hepatitis B and C, metabolic syndromes, alcohol, certain dietary toxins, and aflatoxins [1].

HCC is an overly aggressive malignancy with frequent intrahepatic and extrahepatic metastasis. HCCs are hypervascular malignant tumors and are thought to spread through a hematogenous route, thus leading to widespread metastasis [2]. The prognosis of HCC patients with extrahepatic metastasis is dismal [2-4]. In our practice, we have observed that HCC has the propensity to metastasize to very unusual sites and can sometimes show variable patterns leading to diagnostic difficulty. Given the significant role of recognition of preoperative metastases in selecting treatment options, a better understanding of the metastatic behavior of HCC is exceedingly desirable. In this study of 257 patients, we aim to discuss the unusual sites of HCC metastasis, the various patterns of metastasis, clinicopathological features, and the most common cause of HCC in our population. This study, from a clinicopathological perspective, represents the most comprehensive investigation of metastatic HCC conducted in the past two decades. Its findings offer valuable insights into the clinical implications of this disease, particularly in cases with an unknown medical history or an undiagnosed primary malignancy.

## Materials and methods

We systematically reviewed the available literature using Ovid, the National Digital Library of the Higher Education Commission, BioMed Central, PubMed, and Google Scholar. After completing the online search, full texts were obtained for all relevant articles and studies. Therefore, approval from the Institutional Review Board of Shaukat Khanum Memorial Cancer Hospital and Research Centre was obtained (approval number EX-07-02-22-01).

We retrieved 257 cases of metastatic HCC from the hospital database for the period from 2016 to February 2022 through a systematic search. Inclusion criteria required cases to be diagnosed as metastatic HCC with confirmed immunohistochemical evidence, while exclusion criteria involved cases without verifiable immunohistochemical confirmation.

In this medical research study, a comprehensive histological examination was conducted to assess tissue specimens. The process involved the review of previously prepared tissue slides that were stained with H&E and cut into sections measuring 4-5 µm in thickness. Several specialized laboratory instruments were utilized throughout this procedure, ensuring the accuracy and quality of the examination.

The tissue processing phase was executed using the Leica PELORIS system (Leica Biosystems Nussloch GmbH, Nußloch, Germany), which prepared the tissue samples for further analysis. Subsequently, the samples were embedded in paraffin wax using the ThermoFisher Histostar equipment (Thermo Fisher Scientific Inc., Waltham, MA, USA). Precision in sectioning the tissue was maintained with the Leica RM 2245 microtome (Leica Biosystems Nussloch GmbH). The staining of tissue sections was performed meticulously with the Leica ST 5020 staining apparatus (Leica Biosystems Nussloch GmbH).

Furthermore, the prepared slides were meticulously covered and slipped using the Leica CV 5030 instrument (Leica Biosystems Nussloch GmbH) to protect the tissue sections and facilitate their examination. This comprehensive laboratory workflow ensured the integrity of the tissue specimens and allowed for accurate histological evaluation.

In addition to the laboratory procedures, patient reports were systematically reviewed. To augment this analysis, a thorough assessment of clinical history was conducted to ascertain the cause of the medical condition under investigation. Radiological confirmation, a vital aspect of this study, was sought through various modalities. To collect this information, patients or their attendants were engaged in telephonic conversations. This additional step in data collection provided valuable insights into the patients’ conditions and the diagnostic processes used, further enriching the scope and depth of the research. Statistical analysis was carried out using IBM SPSS Statistics for Windows, Version 20.0 (Released 2011; IBM Corp., Armonk, NY, USA) and Microsoft Excel 2016 (Microsoft Corporation, Redmond, WA, USA), employing these software tools to extract meaningful insights and draw informed conclusions from the gathered data.

History and relevant information were obtained from patients via telephonic conversation or from the hospital’s electronic system in the case of in-house patients. Out of these, 15 patients could not be contacted.

## Results

A total of 257 patients with metastatic HCC were identified in our retrospective database search. Out of these, 209 (81.3%) patients were male, and only 48 (18.7%) were female. The male-to-female ratio is 4.35:1. The minimum age was 12 years, and the maximum was 90 years, with a mean age of 60 years and a standard deviation of 11.76.

**Figure 1:**
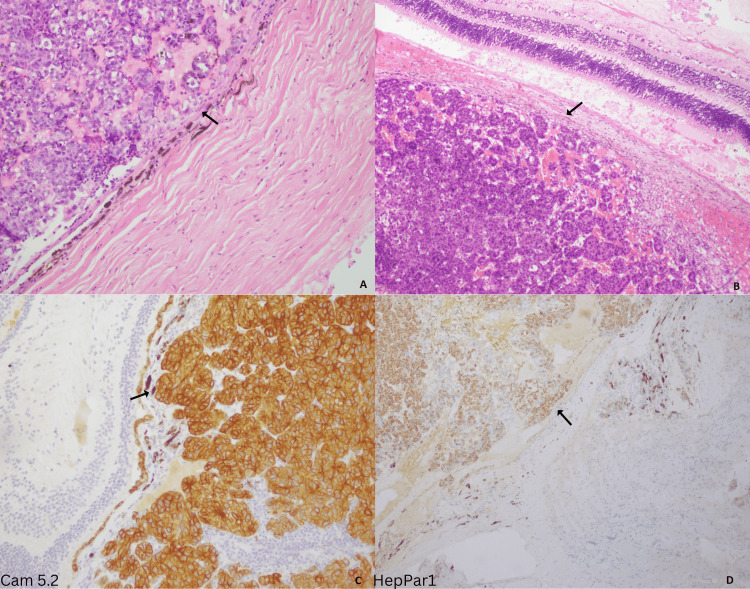
Metastatic tumor to the eye. High power view exhibiting HCC in a nested and trabecular pattern abutting the retina and choroid of the eye (A, B); Cam 5.2 showing diffuse membranous positivity (C); and HepPar1 showing patchy positivity (D). HCC, hepatocellular carcinoma

**Figure 2:**
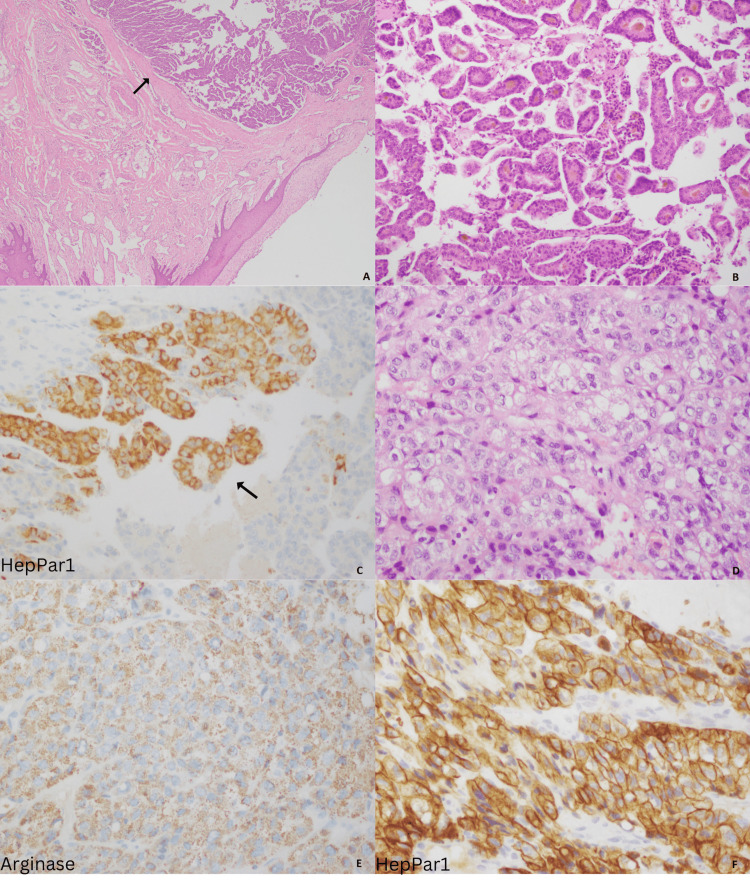
Metastatic tumor to the ring finger and salivary gland. Low power view showing skin of the finger and underlying adnexal structures with a malignant tumor (A); High power view showing a tumor with a pseudoalveolar, trabecular, and nested pattern. Visible bile pigment in lumens also noted (B); HepPar1 showing moderate to strong membranous and cytoplasmic staining (C); Metastatic tumor to parotid gland with clear cell morphology (D); Arginase showing diffuse cytoplasmic staining (E); and HepPar1 showing a diffuse membranous staining pattern (F).

**Figure 3:**
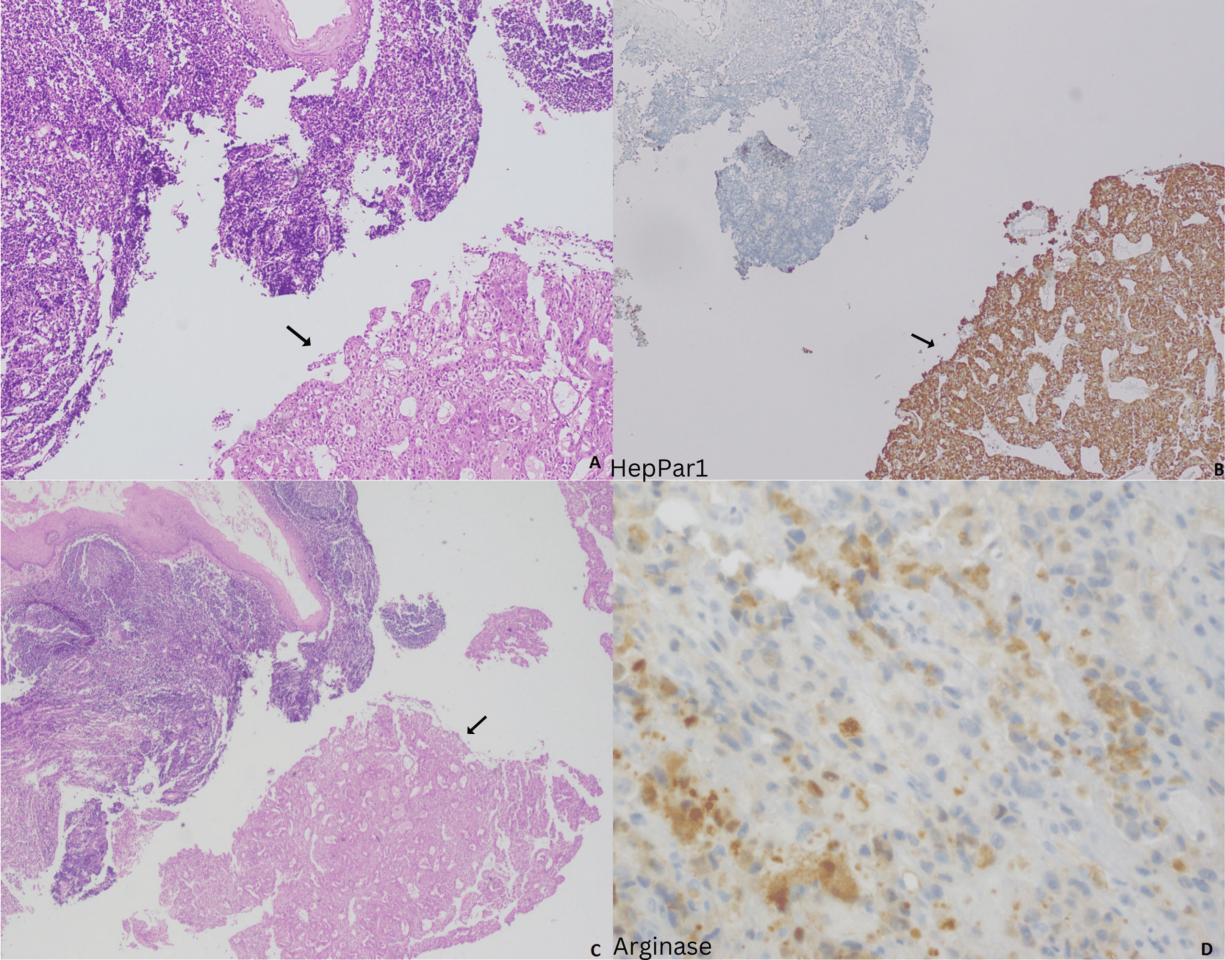
Metastatic tumor to tonsil. High and low power views showing tonsillar crypt lined by squamous epithelium and underlying reactive lymphoid tissue. The lower corner shows a neoplastic proliferation with trabecular and pseudoalveolar pattern (A, C); HepPar1 positivity in tumor (B); and Patchy arginase positivity in tumor (D).

**Figure 4:**
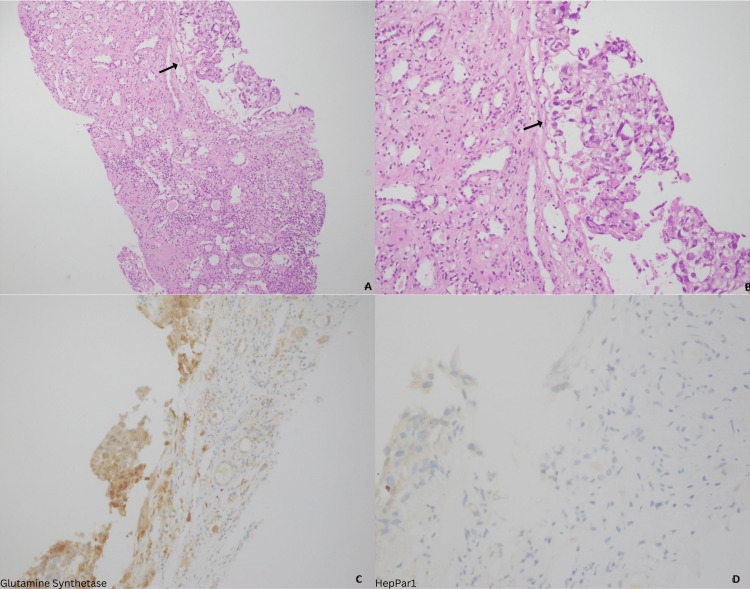
Metastatic tumor to the kidney. Low and high power views showing renal tissue cores with tubules and metastatic HCC with nested and pseudoalveolar pattern (A, B); Glutamine synthetase highlighting tumor cells (C); and HepPar1, in this case, was negative (D). HCC, hepatocellular carcinoma

**Figure 5:**
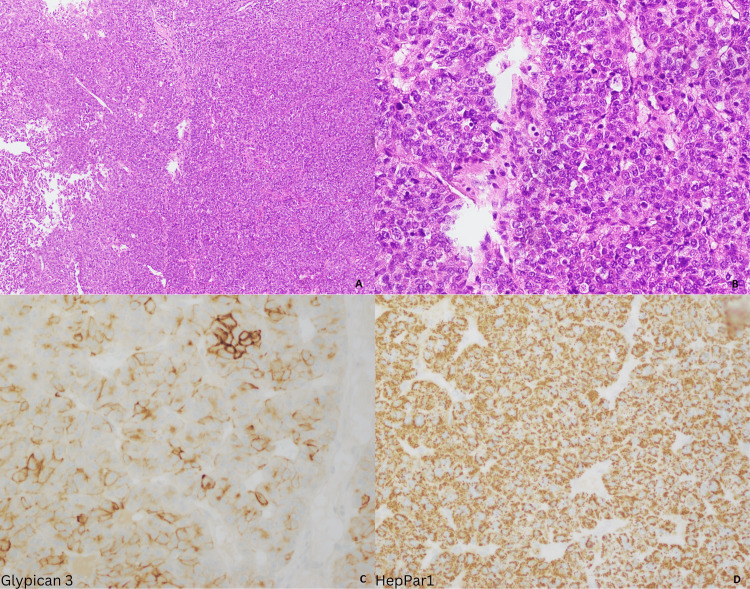
Metastatic tumor to the brain. Low and high power views showing a poorly differentiated tumor with high-grade morphology and solid pattern (A, B); Patchy glypican-3 staining (C); and Diffuse HepPar1 staining (D).

Metastatic sites displayed considerable diversity, with the most prevalent location being bone (52.5%), specifically the vertebral column (24.7%), humerus (7.85%), and femur (6.2%). Among vertebral column metastases, L2 vertebra (3.9%) was the most common. Soft tissue accounted for the second-highest proportion (21.8%), predominantly in the chest wall (6.2%). Additionally, several less common locations were identified through histomorphology and immunophenotypic features (Figures 1-5). Table 1 presents a comprehensive breakdown, including the location, case count, and the corresponding percentage of metastatic sites.

**Table 1:** Distribution of locations, case count, and percentage of metastatic sites.

Location	Number (n)	Percentage (%)
Bone	135	52.5
Vertebral column	63	24.7
Humerus	20	7.85
Femur	16	6.2
Sternum	11	4.2
Iliac bone	10	3.9
Rib	5	1.9
Scapula	3	1.2
Acetabulum	2	0.8
Mandible	2	0.8
Clavicle	1	0.4
Ischium	1	0.4
Tibia	1	0.4
Soft tissue	56	21.8
Chest wall	16	6.2
Shoulder	8	3.1
Scalp	7	2.7
Paraspinal	6	2.3
Abdominal wall	3	1.2
Iliac fossa and pelvic soft tissue	3	1.2
Gluteal mass/buttocks	2	0.8
Axilla	2	0.8
Arm	2	0.8
Orbital	2	0.8
Thigh	2	0.8
Submandibular	1	0.4
Ring finger	1	0.4
Lumbar soft tissue	1	0.4
Head and neck	26	10.1
Brain	9	3.5
Neck and cervical nodes	9	3.5
Gum and buccal mucosa	5	1.9
Eye	1	0.4
Tonsil	1	0.4
Salivary gland	1	0.4
Abdomino-pelvic organs	26	10.1
Adrenal	9	3.5
Omentum	5	1.9
Periportal and para-aortic lymph nodes	5	1.9
Kidney	4	1.6
Spleen	1	0.4
Pancreas	1	0.4
Ovary	1	0.4
Others	14	5.5
Lung	8	3.1
Breast	3	1.2
Retro-peritoneum	2	0.8
Epigastrium	1	0.4
Total	257	100

Table 2 presents a summary of the diverse factors and circumstances linked to the onset of HCC within the examined patients.

**Table 2:** Distribution of HCC etiologies. HCC, hepatocellular carcinoma

Patient groups	Number of patients	Percentage (%)
Hepatitis C	183	71.2
Hepatitis B	14	5.4
Hepatitis B and C	11	4.2
Combined viral hepatitis (total)	208	80.9
Uninfected	24	9.3
Alcohol-induced cirrhosis	1 =	0.4
Undetermined cause	23	8.9
No testing for causative factor	10	3.9

The predominant histological patterns are delineated in Table 3. Approximately 17.8% of cases exhibited a composite histological composition involving nests, trabeculae, sheets, sinusoidal, pseudoalveolar, and pseudopapillary structures. The prevailing cytomorphology across the majority of cases manifested as intermediate to large-sized cells, characterized by moderate to abundant eosinophilic cytoplasm and distinct to prominent nucleoli. Notably, a singular case with metastasis to the parotid gland displayed a markedly clear cytoplasm and nested pattern, introducing diagnostic complexity reminiscent of primary salivary gland neoplasms. Furthermore, a substantial proportion, up to 50%, demonstrated evidence of at least focal bile pigment.

**Table 3:** Summary of histological patterns in metastatic HCC patients. HCC, hepatocellular carcinoma

Histological pattern	Number of patients	Percentage (%)
Pure trabecular	192	74.7
Pure solid sheets of plump cells	12	4.7
Combined nested and trabecular	9	3.5
Mixed patterns (nests, trabeculae, sheets, etc.)	43	17.8
Distinctly clear cytoplasm and nested pattern	1	0.3

This study involved 120 patients (46.7%) with a known history of HCC, but in almost half of these 120 cases, a history was not provided on the request form and later obtained. Out of these 120 patients, 20 were post-liver transplants as a part of treatment for HCC. The history of 15 patients (5.8%) remains unknown due to nonavailability of contact information.

All cases were diagnosed with the help of immunohistochemical stains. Out of 257 cases, 75 cases (29.18%) were diagnosed with the help of a combination of glypican-3 and HepPar1, and 67 cases (26.1%) were diagnosed only based on the positivity of HepPar1. The remaining cases (n = 115) were diagnosed based on combinations of glutamine synthetase, glypican-3, arginase, HepPar1, Cam 5.2, and other cytokeratins. In addition, we did a large battery of other lineage immunohistochemical markers in more than half of the cases owing to odd locations, unknown clinical findings, insufficient history on the request forms, and variable morphological patterns.

As a whole, we performed HepPar1 in a total of 240 cases. It was positive in 205 cases (85.5%), glypican-3 was positive in 113 cases out of 150 cases (75.3%), arginase was positive in 31 out of 42 cases (73.8%), glutamine synthetase was positive in 15 of 30 cases (50%), and Cam 5.2 was positive in 67 of 72 cases (93%).

We also inquired about the radiological modalities utilized for the confirmation of diagnosis. 228 patients (88.5%) were diagnosed on triphasic and biphasic CT scans. In five patients (1.9%), MRI was used as the main diagnostic test. CT and MRI were employed in two patients (0.8%), and PET scan was used in another two patients (0.8%). No confirmatory scans were done in five patients (1.9%), owing to poor socioeconomic status, lack of relevant guidance and knowledge, and sudden deterioration in patients’ health leading to sudden demise, including one patient with metastatic HCC to the posterior chamber of the eye, in whom enucleation was performed on a suspicion of choroidal melanoma. The history of 15 patients (5.8%) is unknown. Out of 257 cases, as many as 122 patients (47.5%) did not have a history of HCC and came to know of the disease after the histopathology report. Most patients were confirmed to have HCC after clinical and radiological workups secondary to suspicion raised in the histopathology report.

## Discussion

HCC, a widespread malignancy in adults, frequently presents with advanced disease, high recurrence, limited treatment options, and elevated metastatic potential, contributing to an overall grim prognosis [5]. In 2018, liver cancer was estimated to rank as the sixth most frequently diagnosed cancer and the fourth leading cause of cancer-related mortality worldwide, accounting for roughly 841,000 new cases and 782,000 deaths annually. The highest incidence rates are predominantly found in regions with lower Human Development Index, such as parts of Northern and Western Africa (e.g., Egypt, Gambia, and Guinea) and Eastern and Southeastern Asia (e.g., Mongolia, Cambodia, and Vietnam) [6]. In Pakistan, the age-standardized incidence rate of HCC is 7.6 per 100,000 individuals per year for males and 2.8 per 100,000 for females [7]. According to the annual cancer registry of Shaukat Khanum Memorial Cancer Hospital and Research Centre, 168 cases of HCC were recorded [8].

Major risk factors leading to HCC include hepatitis C and B, alcoholism, dietary toxins, and metabolic syndrome [5-11]. Other risk factors include non-alcoholic fatty liver disease and certain hereditary diseases such as hemochromatosis, Wilson’s disease, and alpha-1 antitrypsin deficiency [12-17]. These conditions lead to recurrent episodes of inflammation leading to fibrosis, consequently progressing to liver cirrhosis and the development of preneoplastic lesions in the liver [9,10]. Therefore, it is imperative to improve the diagnostic and treatment modalities due to dismal clinical outcomes.

In a study by Schlageter et al., the most common cause was established to be alcohol intake in 68% of patients, followed by viral hepatitis in 33% of patients [4]. However, in our study, 81% of patients had a history of viral hepatitis, predominantly hepatitis C in 71.2%, followed by hepatitis B and combined hepatitis B and hepatitis C infection. Only 0.4% of patients had a history of alcohol intake. This stark contrast can be due to religious, cultural, and social differences. We need to find out the reason. It is widely recognized that alcohol consumption is generally lower in our country compared to Western nations. In a study by Chen et al., viral hepatitis accounted for 88.3% of cases, similar to 81% in our population [13]. However, hepatitis B was the most common in their study (82.2%), whereas it was present in only 5.4% of our cases. This disparity may be attributed to regional and ethnic differences.

Generally, males are affected more, and the average age of presentation is 50-60 years. However, in an extensive series of 195 cases of metastatic HCC in 2018, Chen et al. established that males are affected more by this disease with an M/F ratio of 7.5:1, comparable with our ratio of 4.35:1 [13]. In addition, the mean age in their study was 53 years, compared to the mean age of 60 years in ours.

Overall, these tumors exhibit a range of morphologies, from multinodular to vaguely nodular and from expansive to infiltrative [9,10]. HCCs present various distinct variants at both primary and metastatic sites, adding to the diagnostic challenge. Notable variants include clear cell, sarcomatoid, steatohepatitic, and fibrolamellar types, each displaying diverse histological patterns. According to Rastogi, the predominant pattern in 70% of primary HCCs is trabecular, followed by solid/nested (20%) and pseudo-glandular (10%) [9]. Our metastatic HCC data aligns with this, demonstrating a pure trabecular pattern in 74.7% of cases, while the solid/nested pattern is notably present in only 4.7%. In contrast, Chen et al. reported a solid pattern in 45.1% of patients, diverging from our findings where the most prevalent pattern was pure trabecular (74.7%), with a minimal occurrence of the pure solid pattern (4.7%). These nuanced variations underscore the intricacies of HCC presentation and emphasize the need for comprehensive histological analysis.

Diagnosing HCC is straightforward when the patient’s history is known; however, in the majority of cases with unknown history, the diagnosis becomes challenging due to diverse morphological patterns, absence of typical features, varying degrees of differentiation, and the potential overlap with other tumors exhibiting hepatoid differentiation. These cases were histologically diagnosed based on specific morphological clues, including predominant trabecular pattern, eosinophilic cytoplasm, prominent nucleoli, and bile pigment. In such cases, immunohistochemistry is employed to attain a definitive diagnosis. One of the essential features observed in initial cases was the absence of cytokeratin staining in as many as 62 cases (24.2%), which led to the workup of differential diagnosis of cytokeratin-negative tumors, including HCC. Timek et al. recommended using a panel of three antibodies to establish the primary site as the liver, including arginase-1, glypican-3, and HepPar1 [18]. Yan et al. also recommended arginase as a sensitive HCC marker [19]. Our study endorses these facts, with HepPar1, arginase-1, and glypican-3 positivity seen in 85.5%, 73.8%, and 75.3% cases. Cam5.2 positivity was seen in 93% of cases. However, it is not specific to the hepatic origin and can only be used in the context of positivity with other markers. Another helpful marker is glutamine synthetase, which was positive in 50% of our cases.

HCC has the propensity to metastasize to very odd sites, in our experience. Hematogenous spread is generally more common in HCC than lymphatic spread. In a study by Schlageter et al., the most common site of metastasis was the lung in 75% of patients, followed by the bone and adrenal gland [4]. However, in our study, the most common site was bone (51.7%), followed by chest wall soft tissue, adrenal gland, and brain. Metastasis to the lung was found in only 3.1% of patients. This difference can be because, in our population, lung biopsies are not routinely done when metastasis is suspected, and treatment is done based on radiological findings. However, the abovementioned study is performed on autopsies, profoundly increasing their chances of detecting asymptomatic metastasis.

In the series of 195 cases by Chen, the most common site of metastasis was bone in 57% of patients. Compared to our results, 52.1% of patients had metastatic disease to the bone [13]. Among all bony metastases, the most common site was vertebrae in 53.2% of cases. Our study also revealed the most common site to be the vertebrae, but with a percentage of 24.7%. In contrast to our studies, Wu et al., Yoo et al., Katyal et al., and Natsuizaka et al. [14-17] established the lung as the most common site, and Uchino et al. [3] reported lymph nodes as the most common site. The second common site in our study was soft tissue (21.8%), while in Chen’s study, the second common location of metastasis was the lung (21%). Other sites in Chen’s study were the abdomen, omentum, adrenal gland, soft tissue, pelvis, brain, intestine, stomach, retroperitoneum, kidney, and umbilicus in decreasing order of frequency. Our study also identified metastasis in these locations except for the stomach, intestine, and umbilicus.

The main aim of our study was to establish different locations of HCC metastasis, common causative agents, and their immunohistochemical and histological features. In addition to the findings discussed above, it should be emphasized here that, although some locations are more common than others, HCC has the propensity to metastasize to very odd sites uncommonly. Therefore, it should always be kept in the differential diagnosis when dealing with a metastatic lesion with diversity in morphological patterns and in cases where morphology or immunophenotypic features do not match a primary tumor for that site. This finding is endorsed by our finding of metastatic HCC cases in the parotid gland, eyeball, mandible, ring finger, breast, ovary, and tonsil. Moreover, there are case reports in the literature of metastatic HCC in the heart, nasal cavity, mandible, orbit, parotid, thyroid, and pituitary gland [20-26].

Although we did not do a formal survival analysis in our study due to the insufficient time between our study and the reporting of some cases, we noted that while obtaining relevant clinical information, approximately 80% of patients diagnosed from 2016 to 2018 had passed away due to disease-related problems within three years. Moreover, we also tried to obtain information regarding serum AFP levels for correlation. However, this information was only available for some patients.

## Conclusions

Our study represents the most extensive analysis of the clinicopathological features of metastatic HCC in the past two decades. The primary objective was to explore uncommon metastatic sites, patterns of metastasis, and the clinicopathological characteristics in our population. We identified the trabecular pattern as the most prevalent, with bone (particularly the vertebral column) being the most common metastatic site. Viral hepatitis, notably hepatitis C, was the leading cause of HCC in our population. Tumors were frequently positive for Cam 5.2 and HepPar1, and biphasic and triphasic CT scans were the preferred radiological methods for confirming the hepatic primary. Our findings also highlight histological and immunohistochemical features useful for diagnosing tumors at metastatic sites with an unknown primary.

## References

[1] Yang JD, Hainaut P, Gores GJ, Amadou A, Plymoth A, Roberts LR (2019). A global view of hepatocellular carcinoma: trends, risk, prevention and management. Nat Rev Gastroenterol Hepatol.

[2] Uka K, Aikata H, Takaki S (2007). Clinical features and prognosis of patients with extrahepatic metastases from hepatocellular carcinoma. World J Gastroenterol.

[3] Uchino K, Tateishi R, Shiina S (2011). Hepatocellular carcinoma with extrahepatic metastasis: clinical features and prognostic factors. Cancer.

[4] Schlageter M, Quagliata L, Matter M, Perrina V, Tornillo L, Terracciano L (2016). Clinicopathological features and metastatic pattern of hepatocellular carcinoma: an autopsy study of 398 patients. Pathobiology.

[5] Agni RM (2017). Diagnostic histopathology of hepatocellular carcinoma: a case-based review. Semin Diagn Pathol.

[6] Bray F, Ferlay J, Soerjomataram I, Siegel RL, Torre LA, Jemal A (2018). Global cancer statistics 2018: GLOBOCAN estimates of incidence and mortality worldwide for 36 cancers in 185 countries. CA Cancer J Clin.

[7] Hafeez Bhatti AB, Dar FS, Waheed A, Shafique K, Sultan F, Shah NH (2016). Hepatocellular carcinoma in Pakistan: national trends and global perspective. Gastroenterol Res Pract.

[8] Annual Cancer Registry Report-2021: Shaukat Khanum Memorial Cancer Hospital & Research Center, Pakistan. https://shaukatkhanum.org.pk/wp-content/uploads/2022/05/Annual-Cancer-Registry-Report-2021.pdf.

[9] Rastogi A (2018). Changing role of histopathology in the diagnosis and management of hepatocellular carcinoma. World J Gastroenterol.

[10] Schlageter M, Terracciano LM, D'Angelo S, Sorrentino P (2014). Histopathology of hepatocellular carcinoma. World J Gastroenterol.

[11] El Jabbour T, Lagana SM, Lee H (2019). Update on hepatocellular carcinoma: pathologists' review. World J Gastroenterol.

[12] Quaglia A (2018). Hepatocellular carcinoma: a review of diagnostic challenges for the pathologist. J Hepatocell Carcinoma.

[13] Chen D, Li Z, Song Q, Qian L, Xie B, Zhu J (2018). Clinicopathological features and differential diagnosis of hepatocellular carcinoma in extrahepatic metastases. Medicine (Baltimore).

[14] Wu W, He X, Andayani D (2017). Pattern of distant extrahepatic metastases in primary liver cancer: a SEER based study. J Cancer.

[15] Natsuizaka M, Omura T, Akaike T (2005). Clinical features of hepatocellular carcinoma with extrahepatic metastases. J Gastroenterol Hepatol.

[16] Yoo DJ, Kim KM, Jin YJ (2011). Clinical outcome of 251 patients with extrahepatic metastasis at initial diagnosis of hepatocellular carcinoma: does transarterial chemoembolization improve survival in these patients?. J Gastroenterol Hepatol.

[17] Katyal S, Oliver JH 3rd, Peterson MS, Ferris JV, Carr BS, Baron RL (2000). Extrahepatic metastases of hepatocellular carcinoma. Radiology.

[18] Timek DT, Shi J, Liu H, Lin F (2012). Arginase-1, HepPar-1, and Glypican-3 are the most effective panel of markers in distinguishing hepatocellular carcinoma from metastatic tumor on fine-needle aspiration specimens. Am J Clin Pathol.

[19] Yan BC, Gong C, Song J (2010). Arginase-1: a new immunohistochemical marker of hepatocytes and hepatocellular neoplasms. Am J Surg Pathol.

[20] Masci G, Magagnoli M, Grimaldi A, Covini G, Carnaghi C, Rimassa L, Santoro A (2004). Metastasis of hepatocellular carcinoma to the heart: a case report and review of the literature. Tumori.

[21] Liu CY, Chang LC, Yang SW (2011). Metastatic hepatocellular carcinoma to the nasal cavity: a case report and review of the literature. J Cancer Sci Ther.

[22] Chin A, Liang TS, Borislow AJ (1998). Initial presentation of hepatocellular carcinoma as a mandibular mass: case report and review of the literature. Oral Surg Oral Med Oral Pathol Oral Radiol Endod.

[23] Oida Y, Ohtani Y, Dowaki S (2006). Hepatocellular carcinoma metastatic to the orbit: a case report. Tokai J Exp Clin Med.

[24] Yu YD, Kim DS, Jung SW, Lee JH, Chae YS, Suh SO (2013). Metastatic hepatocellular carcinoma to the parotid gland: case report and review of the literature. Int J Surg Case Rep.

[25] Liang HH, Wu CH, Tam KW, Chai CY, Lin SE, Chen SC (2007). Thyroid metastasis in a patient with hepatocellular carcinoma: case report and review of literature. World J Surg Oncol.

[26] Karamouzis MV, Melachrinou M, Fratzoglou M, Labropoulou-Karatza Ch, Kalofonos HP (2003). Hepatocellular carcinoma metastasis in the pituitary gland: case report and review of the literature. J Neurooncol.

